# High transconjugation efficiency of fusion plasmid pNDM_KPC in carbapenem-resistant *Citrobacter freundii* and its formation driven by IS*26*-mediated integration

**DOI:** 10.1128/spectrum.00905-25

**Published:** 2025-08-14

**Authors:** Yuxiaoting Li, Xinhong Han, Xiangping Shi, Caiping Mao, Ting Yu, Yufeng Zhu, Shengjun Wu, Youping Huang, Yunsong Yu, Ying Fu

**Affiliations:** 1 Department of Clinical Laboratory, Sir Run Run Shaw Hospital, Zhejiang University School of Medicine 26441 , Hangzhou, Zhejiang Province, China; 2 Key Laboratory of Precision Medicine in Diagnosis and Monitoring Research of Zhejiang Province, Hangzhou, Zhejiang Province, China; 3 Zhejiang Provincial Engineering Research Center of Innovative Instruments for Precise Pathogen Detection, Hangzhou, Zhejiang Province, China; 4 Department of Clinical Laboratory, Zhejiang Cancer Hospital, Hangzhou Institute of Medicine (HIM), Chinese Academy of Sciences 631027 , Hangzhou, Zhejiang Province, China; 5 Department of Clinical Laboratory, Shaoxing Second Hospital 74622, Shaoxing, Zhejiang Province, China; 6 Department of Open Laboratory Medicine, Hangzhou Xixi Hospital, School of Medicine, Zhejiang University 12377, Hangzhou, Zhejiang Province, China; 7 Department of Clinical Laboratory, The First Peoples' Hospital of Fuyang 682994 , Hangzhou, Zhejiang Province, China; 8 Department of Infectious Diseases, Sir Run Shaw Hospital, Zhejiang University School of Medicine 26441 , Hangzhou, Zhejiang Province, China; 9 Key Laboratory of Microbial Technology and Bioinformatics of Zhejiang Province, Hangzhou, Zhejiang Province, China; 10 Regional Medical Center for National Institute of Respiratory Diseases, Sir Run Run Shaw Hospital, Zhejiang University School of Medicine 26441 , Hangzhou, Zhejiang Province, China; Institut National de Santé Publique du Québec, Sainte-Anne-de-Bellevue, Québec, Canada

**Keywords:** Carbapenem-resistant, *Citrobacter freundii*, pNDM_KPC, NDM-1, KPC-2, IS*26*, transconjugation efficiency, integration, *IntI*

## Abstract

Carbapenem-resistant *Enterobacteriales* (CRE) are increasingly recognized as reemerging nosocomial pathogens due to the dissemination of dual carbapenemase production. This study investigates the coexistence of *bla*_KPC-2_ and *bla*_NDM-1_ in CRE isolates, harbored on plasmids either separately and integratively, and examines the underlying mechanisms of plasmid formation and resistance dissemination. Two *Citrobacter freundii* isolates co-producing *Klebsiella pneumoniae* carbapenemase (KPC) and New Delhi metallo-beta-lactamase (NDM) carbapenemases were analyzed. S1-PFGE, Southern blot, and mating conjugation experiments were conducted to determine gene location, transferability, and transconjugation efficiency. Whole-genome sequencing (WGS) was used to elucidate plasmid evolution mechanisms. Specific PCR followed by sequencing was performed to identify hotspot integration sites. Experiments of checkerboard microdilution and time-kill were completed to test the treatment option based on aztreonam. Three plasmids were identified in *C. freundii,* pNDM, pKPC, and a fusion plasmid (pNDM_KPC) co-harboring *bla*_KPC-2_ and *bla*_NDM-1_. All plasmids exhibited transconjugation capability, with significant differences in transfer efficiency (*P* < 0.05). pKPC demonstrated the highest efficiency (ranging from 10⁻¹ to 10⁻^2^), followed by pNDM_KPC (10⁻^4^ to 10⁻^6^) and then pNDM (around 10⁻⁸). IS*26*, located downstream of the *intI* site, was identified as a critical hotspot mediating the disintegration and integration of pNDM_KPC. Synergy effect and bactericidal activity were observed on aztreonam plus ceftazidime/avibactam or meropenem/vaborbactam or imipenem/relebactam. The presence of CRE isolates harboring fusion plasmid pNDM_KPC would pose a significant threat in nosocomial infections and should arise wide attention. IS*26* downstream of IntI would be the hotspot driving the integration state of pNDM_KPC and contributing to complex resistance mechanisms.

**IMPORTANCE:**

The global rise of carbapenem-resistant *Enterobacterales* (CRE) poses a critical threat to public health. Our study identifies *Citrobacter freundii* strains co-harboring three distinct carbapenemase-encoding plasmids: pNDM, pKPC, and a novel fusion plasmid (pNDM_KPC). We reveal significant differences in conjugation efficiency among these plasmids, with the fusion plasmid demonstrating a moderate yet concerning ability to spread dual carbapenemase genes. This finding highlights the fusion plasmid as a potential vector for the co-dissemination of *bla*_NDM-1_ and *bla*_KPC-2_, emphasizing the need for enhanced surveillance. Furthermore, we identify the IS*26* element downstream of the *intI* site as a potential recombination hotspot driving plasmid fusion. These insights deepen our understanding of carbapenemase gene transmission and call for global attention to the fusion plasmid-mediated resistance spread.

## INTRODUCTION

Carbapenem-resistant *Enterobacterales* (CRE) pose a severe threat to global health due to limited treatment options and high level of morbidity and mortality (1). Of the different classes of carbapenemases, the *Klebsiella pneumoniae* carbapenemase (KPC) and New Delhi metallo-beta-lactamase (NDM) types are predominant carbapenemases in CRE isolates and have disseminated both globally and nationally (2). To those infections caused by CRE isolates producing either KPC or NDM, the treatments of ceftazidime/avibactam (CZA) or aztreonam (ATM) in clinical would be effective, whereas monotherapy would fail if isolates produce dual carbapenemases (3). What is even more concerning is that an increasing trend of CRE strains producing dual carbapenemases, typically KPC-2 and NDM-1, has been reported widely over the past 5 years (4–8).

The annual incidence of KPC-2-NDM-1-producing carbapenem-resistant *Klebsiella pneumoniae* in China increased from 0.28% in 2016 to 0.58% in 2017 (4). Additionally, previous reports have identified *C. freundii* strains producing dual carbapenemases isolated from both environmental samples and patients (6, 7). In the wake of this pressing phenomenon, a deeper understanding of the resistance characteristics, genetic factors, and fitness costs involved could shed light on the persistence and propagation of these resistant bacteria.

Homologous recombination is considered an important mechanism for bacteria to acquire novel phenotypes or integrate multiple resistance genes (9, 10). Literature researches reported that the KPC plasmids have increased in size over time, and the types of plasmids, including large plasmids, continuously acquire more exogenous genes to enhance their potential (10, 11). NDM plasmids are considered relatively stable, with most reported to be less than 100 kb in size according to multiple studies (12, 13). Sub-groupings of *Inc*M2 plasmids encoding *bla*_NDM-1_ were detected from five different species of *Enterobacterales* (*Citrobacter spp*., *Enterobacter cloacae*, *Escherichia coli*, *Klebsiella pneumoniae,* and *Klebsiella oxytoca*) (14). From the initial reports until now, there has been a certain proportion of *Inc*X3, 50 kb small plasmids stably maintained in hospital-isolated strains (13, 15), exhibiting evolutionary characteristics different from KPC plasmids.

This study initially discovered two strains of *C. freundii* carrying both *bla*_KPC-2_ and *bla*_NDM-1_, exhibiting a pan-drug-resistant profile. The two resistant genes coexisted either on separate plasmids or integrated into a single plasmid. By comparing the transfer efficiency of plasmids in transconjugation experiments, our results reveal a considerable threat of CRE producing KPC and NDM in infection control. Further, we seek to outline the potential routes of horizontal gene transfer and plasmid evolutionary characteristics leading to the spread of resistance. Finally, we explored the feasibility of antibiotic combinations as potential therapeutic strategies against these pan-drug-resistant strains.

## MATERIALS AND METHODS

### Data source, species identification, and antimicrobial susceptibility testing (AST)

Two *C. freundii* strains, termed *C. freundii* ZGX and *C. freundii* WMY, were collected from Sir Run Run Shaw Hospital (SRRSH) in Zhejiang Province, China, in 2019. Species identification of bacteria was confirmed using matrix-assisted laser desorption/ionization time-of-flight mass spectrometry (MALDI-TOF MS, bioMérieux, France). The MICs of ceftazidime/avibactam, colistin, and tigecycline were determined using the broth dilution method according to CLSI M07 guideline (16). AST of other antibiotics was conducted on the VITEK two compact system (bioMérieux, France) following the manufacturer’s instructions. Results were interpreted based on the MIC breakpoints of CLSI M100 (17), with *Escherichia coli* ATCC 25922 and *Staphylococcus aureus* ATCC 25923 serving as a quality control.

### Detection of carbapenem-resistant phenotypes and confirmation of carbapenemase genes

The *in vitro* multiplex immunoassay (CGI test, Gold Mountain River Tech Development Company, Beijing, China) was used for the differentiation of five common carbapenemase families (KPC, OXA-48-like, VIM, IMP, and NDM) directly from bacterial colonies (18, 19). Subsequently, PCR followed by sequencing was used to confirm the presence of carbapenemase genes like *bla*_KPC_, *bla*_NDM_, *bla*_IMP_, *bla*_VIM_ and *bla*_OXA-48_, as reported previously (20, 21).

### Identification of plasmids harboring *bla*_KPC_ and *bla*_NDM_

Pulse-field gel electrophoresis (PFGE) was utilized to generate plasmid fingerprints after digestion with S1 restriction enzyme. Southern blotting experiment with *bla*_KPC_ and *bla*_NDM_ as probes was then performed to determine the location of the carbapenemase genes, following established protocols (22).

PCR followed by sequencing was used to detect the presence of pNDM, pKPC, and a fusion plasmid (pNDM_KPC) co-harboring *bla*_KPC-2_ and *bla*_NDM-1_. The primers are shown in Table S1 and Fig. S3.

### Conjugation assay and transconjugation efficiency

Using *E. coli* J53 (NaN3^R^) and *E. coli* EC600 (rifampicin resistance, RIF^R^) as recipient strains, filter mating experiments were conducted to investigate the transconjugability of the plasmid (22). Double-selective plates containing NaN3 (200 µg/mL) or RIF (200 µg/mL) plus CZA (16 µg/mL), NaN3 (200 µg/mL) or RIF (200 µg/mL) plus ATM (4 µg/mL), and NaN3 (200 µg/mL), or RIF (200 µg/mL) plus gentamicin (GEN, 8 µg/mL) were used to select transconjugants containing different plasmids of *bla*_NDM_, *bla*_KPC_ and *bla*_NDM_ plus *bla*_KPC_, respectively. Positive colonies growing on the selective plates were selected for confirmation of carbapenemase genes and pNDM_KPC with PCR followed by sequencing.

Conjugation efficiencies between the donors and *E. coli* J53 recipients were calculated by dividing the number of transconjugants (CFU/mL) by the number of donor or recipient cell (CFU/mL), multiplied by 100%. If the mixture did not show colony growth on the double-selection plate, take the detection limit (<20 CFU/mL) and use the minimum conjugate count of 19 for further calculation. ASTs were then completed on the transconjugants being positive on carbapenemase genes as previously reported or primers of fusion plasmid of pNDM_KPC by PCR (23).

Three technical triplicates and four biological triplicates were performed for each mating experiment. The difference between target groups was estimated by GraphPad Prism version six using one-way analysis of variance (ANOVA) followed by Tukey’s multiple comparisons tests, considering values with an adjusted *P* value lower than 0.05 as significant.

### Whole-genome sequencing (WGS) and data analysis

Genomic DNA extraction was performed with a QIAamp DNA Mini Kit (Qiagen Valencia, CA). An Illumina HiSeq X 10 platform (Illumina, San Diego, CA) and a MinION device (Oxford Nanopore Technologies Inc, UK) were used for WGS. RAST (https://rast.nmpdr.org/rast.cgi) and Prokka 1.11 were used to generate the complete genome sequence and for gene annotation, respectively. The online tools CGE (https://genepi.food.dtu.dk/resfinder) and BacAnt (https://bacant.net) were used to identify the acquired antibiotic resistance genes, plasmid incompatibility types, and mobile elements, respectively. Conjugation transfer capability was predicted by oriTfinder2 (https://bioinfo-mml.sjtu.edu.cn/oriTDB2/). A graphic map was generated by the Proksee server (24). Sequence comparisons were performed using BLASTn v2.4.0 and visualized using Proksee (https://proksee.ca/).

The nucleotide sequences were submitted to the NCBI database with BioSample Accession SAMN35300165 and SAMN35301134.

### Stability of plasmid in transconjugants during serial passage

Transconjugants of *E. coli* J53 (pZGQ_NDM_KPC) and *E. coli* J53 (pWYM_NDM_KPC) were streaked on double antibiotic plates containing NaN3 200 µg/mL and CZA 256/4 µg/mL. Passage experiments were then completed under conditions of no antibiotic selection as previously reported (23). Briefly, three single colonies were picked out into 2 mL antibiotic-free Luria-Bertani (LB) broth and cultured under shaking condition (200 rpm) at 37°C. Overnight cultures were used for inoculation at a 1:1,000 dilution daily for a total of 10 days. Cultures were serially diluted using PBS and grown on Mueller-Hinton (MH) agar with no antibiotics every 5 days. Subsequently, 50 clones were randomly selected and transferred to MH plates supplemented with 100 µg/mL ampicillin for PCR verification of resistance genes. Three technical replicates and four biological replicates were performed for each passage experiment.

### Growth rate determination

To investigate the fitness cost of transconjugants compared to the donor, growth rate experiments were conducted in an antibiotic-free environment as previously described (25). Growth curves were estimated using GraphPad Prism version 6, employing one-way analysis of variance (ANOVA) followed by Tukey’s multiple comparisons test, considering values with an adjusted *P* value lower than 0.05 as significant. Four biological replicates were performed for each passage experiment.

### Checkerboard microdilution method and time-kill experiment

To explore the antibiotic effect *in vitro* and to provide anecdotal clinical data on KPC-2 and NDM-1 co-producing *Enterobacterales* infection, combination experiments based on aztreonam (ATM) were conducted on *C. freundii* WYM, *C. freundii* ZGQ, and *E. coli* J53 (pWYM_ NDM_KPC) by using the checkerboard method and time-kill experiment. The MICs of ATM, CZA, meropenem/vaborbactam (MEV), and imipenem/relebactam (IMR) were tested. Then, the checkerboard microdilution method was applied to determine the combination activity of ATM plus CZA, ATM plus MEV, and ATM plus IMR *in vitro*, and the combination effects were interpreted as the previous report (26).

The time-kill experiment was completed to detect bactericidal efficiency of combination therapy *in vitro*. It was performed according to CLSI guidelines M26 with minor modifications (27). Briefly, the bacterial suspensions were adjusted to ~10^6^ CFU/mL in 2 mL cation-adjusted Mueller-Hinton broth (CAMH) initially, and shaken under the condition of 37°C and 200 rpm with different antibiotic selection. The selected concentrations of ATM, CZA, MEV, and IMR were fixed at 16, 16/4, 16/8, and 4/4 µg/mL, respectively. By using bacterial suspension without antibiotic as the experiment blank control, a dual combination of ATM plus ceftazidime/avibactam, ATM plus MEV, or ATM plus IMR was completed at the fixed concentration. Colony counts were calculated by microdilution method at the time points of 0, 2, 4, 8, 10, and 24 h. The theoretical lower limit of quantification for colony counts was 25 CFU/mL. Time-kill curves were generated by plotting the average colony number, and the time-kill effects were compared between the single agents alone and in combination. Bactericidal activity and synergy were defined as the previously reported (28).

## RESULTS

### Dual carbapenemase production in *C. freundii*

The two carbapenemase-producing *C. freundii* isolates were separated from two inpatients suffering from a breast tumor and respiratory failure, respectively (Table S2). Both isolates exhibited pan-drug resistance to all antibiotics tested with only colistin and tigecycline exhibiting *in vitro* effectiveness (Table S3).

**Fig 1:**
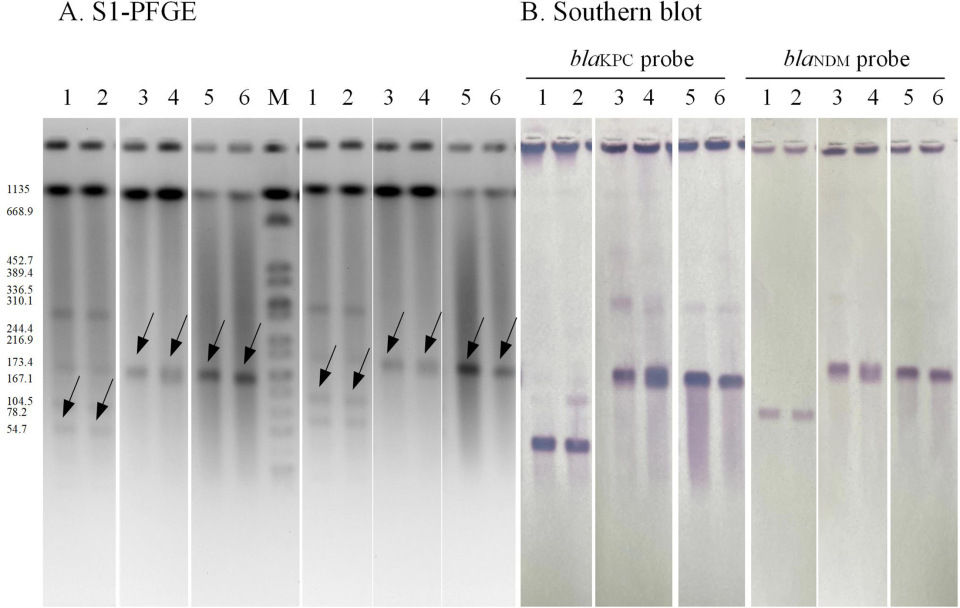
Result of S1-PFGE and southern blot. (A) S1-digested plasmid DNA. (B) Southern blot hybridization of *C. freundii* with *bla*_KPC-2_ or *bla*_NDM-1_. the black arrows present bands of plasmids hybridized with *bla*_NDM_ probe or *bla*_kpc_ probe, respectively. 1, *C. freundii* WYM; 2, *C. freundii* ZGQ: 3, *E. coli* J53 (pWYM _NDM_KPC); 4, *E. coli* J53 (pZGQ_NDM _KPC); 5, *E. coli* EC600 (pWYM_NDM_ KPC); 6, *E. coli* EC600 (pZGQ_NDM_KPC). M, *Salmonella enterica*, serotype Braenderup H9812 digested by Xbal restriction enzyme was used as a marker.

Both the isolates were positive for KPC and NDM carbapenemases (Fig. S1). The S1-PFGE and Southern blot revealed that *bla*_KPC-2_ and *bla*_NDM-1_ were located on plasmids approximately 50 and 100 kb in size, respectively (Fig. 1). Interestingly, the PCR amplification fragments targeting pNDM_KPC fusion plasmid were positive in both isolates and confirmed by sequencing, indicating the presence of a fusion plasmid co-harboring *bla*_KPC-2_ and *bla*_NDM-1_.

### Transconjugability of plasmids

The conjugation assays confirmed the successful transfer of the plasmids harboring *bla*_KPC-2_ and *bla*_NDM-1_ to the recipient cells. By using *C. freundii* WYM as a donor cell, four transconjugants were obtained, including *E. coli* J53 (pWYM_NDM), *E. coli* J53 (pWYM_KPC), *E. coli* J53 (pWYM_NDM_KPC), and *E. coli* EC600 (pWMY_NDM_KPC) (Fig. 1). When the *C. freundii* ZGQ was used as a donor, three transconjugants like *E. coli* J53 (pZGQ_KPC), *E. coli* J53 (pZGQ_NDM_KPC), and *E. coli* EC600 (pZGQ_NDM _ KPC) were selected successfully (Fig. 1, Fig. S1).

Further confirmation by S1-PFGE and Southern blot showed that the transconjugants carried double-positive carbapenemase genes. The two genes were located on the same plasmid in size from 173.4 kb to 244.4 kb in size (Fig. 1). All transconjugants demonstrated close resistance profiles to β-lactams, β-lactams/β-lactamase inhibitor combinations, and carbapenems, with variable resistance to ATM, aminoglycosides, quinolone, and other antibiotic classes (Table S4).

The efficiency of transconjugation was then calculated, revealing significant differences in transfer rates among the different target plasmid groups (Table 1). All plasmids were capable of transconjugation, with statistically significant differences in transfer efficiency (*P* < 0.05). The pKPC demonstrated the highest efficiency (ranging from 10^−1^ to 10^−2^), followed by pNDM_KPC (10⁻^4^ to 10⁻^6^) and then pNDM (around 10⁻⁸) (Table 1).

**TABLE 1:** Transconjugation efficiencies of plasmids from donor to *E. coli* J53^*a*^^,^^*e*^

Target transconjugant group	*C. freundii* WYM ($\bar{x}\pm s$)		*C. freundii* ZGQ ($\bar{x}\pm s$)	
	Donor cell^*b*^	Recipient cell^*c*^	Donor cell^*b*^	Recipient cell^*c*^
*E. coli* J53 (pKPC)	8.30 × 10^–2^ ± 0.95 × 10^–2^	4.40 × 10^–1^ ± 1.54 × 10^–1^	7.45 × 10^–1^ ± 5.18 × 10^–1^	3.44 ± 1.50
*E. coli* J53 (pNDM_KPC)	6.40 × 10^–6^ ± 1.08 × 10^–6^	9.20 × 10^–6^ ± 1.15 × 10^–6^	1.50 × 10^–5^ ± 1.28 × 10^–5^	1.13 × 10^–4^ ± 1.08 × 10^–4^
*E. coli* J53 (pNDM)	6.30 × 10^–8 *d*^	9.00 × 10^–8 *d*^	NA	NA
*P* value	<0.05	<0.05	<0.05	<0.05

^*a$\bar{x}$*^ *,* average; *s,* standard deviation.

^*b*^ The transconjugation efficiency is calculated by the number of the tranconjugants divided by the number of the donor cells.

^*c*^ The transconjugate efficiency is calculated by the number of the transconjugants divided by the number of the recipient cells.

^*d*^ None positive colony is gained and the number is calculated by using the minimum conjugate count of 19, and standard deviation is not calculated.

^*e*^ NA, none positive transconjugant of *E. coli* J53 (pNDM) is obtained when using *C. freundii* ZGQ as a donor. The *P* value was calculated based on the results of pairwise comparisons between the target transconjugant groups, and when it is less than 0.05, it indicates a statistically significant difference.

### Genomic information

**Fig 2:**
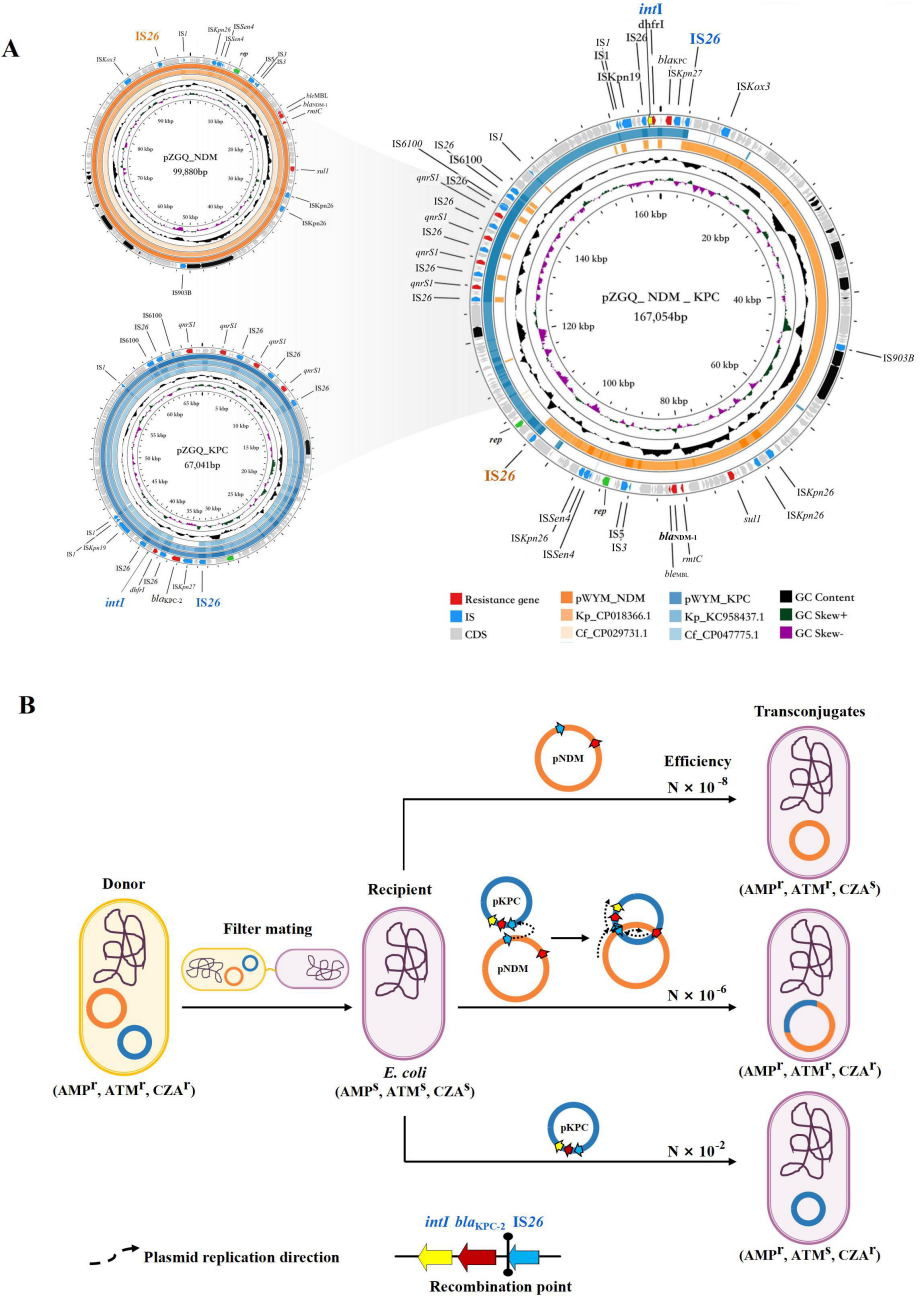
Plasmid structure and transconjugation pattern. (A) Genomic structures of pZGQ_KPC and pZGQ_NDM, and their comparison with pZGQ_NDM_KPC. (B) The transconjugation pattern with filter mating experiment. N ranges from one to ten.

The two *C. freundii* exhibited high similarity over 99%, both belonging to ST118, each carrying one 5.1 Mb chromosome and three resistance plasmids (pNDM, pKPC, and pNDM_KPC) (Fig. 2, Table S4). It harbored multiple resistance genes (*aac(6')-Ib-cr_1*, *aadA1*, *dfrA1*, *qnrS1*, *arr-3*, *bla*_CMY-48_, and *catA1*) in chromosome, and an additional *aac(6')-Ib3* was carried by *C. freundii* ZGQ. The resistance plasmids varied in size and composition. The *bla*_NDM-1_ was located on a 99 kb *Inc*FB and *Inc*FIIY type plasmid, together with *rmtC* and *sul1*, while a 67 kb *Inc*N type plasmid carried *bla*_KPC-2_, *dfrA14,* and four copies of *qnrS1*. The pKPC plasmid contained complete transfer elements, including oriT, relaxase, T4CP, and T4SS, whereas the pNDM plasmid lacked the oriT region (Fig. S2). Additional resistance genes (*terW* and *terZ*) were located on a 289 kb *Inc*HI2 type plasmid. The fusion plasmids, pNDM_KPC, contained both *bla*_NDM-1_ and *bla*_KPC-2_ identified as well.

### The pNDM_KPC was co-integrated by pNDM and pKPC driven by IS*26*-mediated integration at *IntI* sites

Genomic comparisons revealed the pNDM_KPC, showing high similarity to both pNDM and pKPC, was formed through IS*26*-mediated integration. (Fig. 2) The pNDM contained a single copy of IS*26*, while the pKPC harbored nine copies. The IS*26* sequence adjacent to the *bla*_KPC-2_ gene on the pKPC plasmid facilitated its integration with the IS*26* sequence on the pNDM plasmid, with the assistance of the integron *IntI* located next to the *bla*_KPC-2_ gene (Fig. S3). These findings suggest that the integron played a crucial role in facilitating the recombination of the fusion plasmid. This event produced a hybrid plasmid whose size represented the cumulative sum of the two original plasmids remaining all the resistance genes from both sources. This IS*26*-mediated fusion highlights the dynamic nature of plasmid evolution and the potential for the rapid dissemination of multidrug resistance.

### Stably inherited and fitness cost of the hybrid carbapenem-resistant plasmid

To ascertain the stability of the carbapenemase genes in the transconjugants *E. coli* J53 (pZGQ_NDM_KPC) and *E. coli* J53 (pWYM_NDM_KPC), serial passage experiments under antibiotic-free conditions were conducted. The carbapenemase genes remained stable (stability = 100.0%) after 5 and 10 days of passage in the antibiotic-free conditions, indicating that the fusion plasmid could be stably inherited. Strains related to the passage experiments are listed in Table S5.

Growth rate analysis revealed that the transconjugants grew at significantly slower rates compared with the recipient strain *E. coli* J53 (Fig. S4), indicating the fitness cost incurred by the host on harboring carbapenem-resistant plasmids. The plasmid harboring *bla*_KPC-2_ imposed the least fitness cost, while capturing both carbapenem-resistance genes incurred the highest fitness burden. Moreover, the growth rate decreased with the increasing number of carbapenem resistance genes. After 10 days passage, the growth rates of the strains P10 of *E. coli* J53 (pZGQ_NDM_KPC) and P10 of *E. coli* J53 (pWYM_NDM_KPC) were diminished compared with *E. coli* J53 (pZGQ_NDM_KPC) and *E. coli* J53 (pWYM_NDM_KPC).

### Synergy and bactericidal activity of therapy based on ATM

Checkerboard microdilution assays were performed based on ATM combination with CZA, MEV, and IMR. The isolates (*C. freundii* WYM, *C. freundii* ZGQ, and *E. coli* J53 [pWYM_NDM_KPC]) are all resistant to ATM, CZA, MEV, and IMR, individually. However, the combination exhibited strong synergy, restoring all MICs towards these four antibiotics to susceptible levels (Table 2).

**TABLE 2:** Result of checkerboard experiment based on aztreonam combination^*a*^

Strain name	MICs of single antibiotic (μg/mL)				Checkerboard microdilution result											
					ATM + CZA				ATM + MEV				ATM + IMR			
	ATM	CZA	MEV	IMR	MIC of ATM (μg/mL)	MIC of CZA (μg/mL)	FICI	Combination effect	MIC of ATM (μg/mL)	MIC of MEV (μg/mL)	FICI	Combination effect	MIC of ATM (μg/mL)	MIC of IMR (μg/mL)	FICI	Combination effect
*C. freundii* WYM	**>2,048**	**>2,048**	**256**	**64**	0.25	0.06	0.0003	Synergy	1	0.25	0.0011	Synergy	0.5	0.125	0.0019	Synergy
*C. freundii* ZGQ	**>2,048**	**>2,048**	**128**	**64**	0.25	0.06	0.0003	Synergy	0.5	0.25	0.0020	Synergy	0.5	0.125	0.0019	Synergy
*E. coli* J53 (pZGQ_NDM_KPC)	**512**	**>2048**	**64**	**64**	0.125	0.06	0.0004	Synergy	0.125	0.25	0.0036	Synergy	0.5	0.25	0.0024	Synergy

^*a*^ ATM, azteonam; CZA, ceftazidime/ avibactam; MEV, meropenem/vaborbactam; IMR, imipenem/relebactam. If the measured MIC exceeds the detection value, the maximum value is used for calculation of the FICI values. Numbers in bold indicate resistance according to CLSI M100 guidelines.

**Fig 3:**
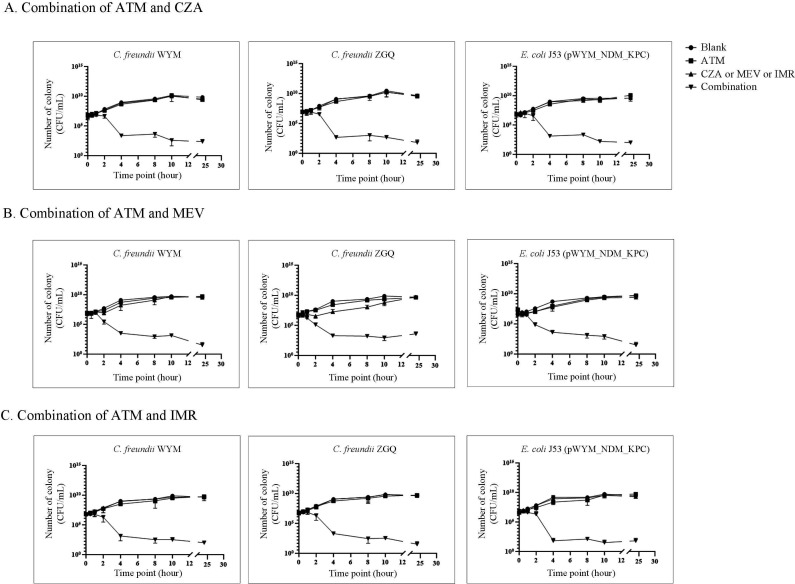
Time–kill curves of different antibiotics combinations against strains studied (A–C). ATM, aztreonam; CZA, ceftazidime/avibactam; MEV, meropenem/vaborbactam; IMR, imipenem/relebactam. Blank means no antibiotic added. The concentration of ATM is 16 µg/mL, IMR 4/4 µg/mL, MEV 16/8 µg/mL, and CZA 16/4 µg/mL.

In time-kill assays, synergy was defined as a ≥2 log_10_ CFU/mL increase in bacterial killing compared with the most active single agent. Bactericidal activity was defined as a ≥3 log_10_ CFU/mL reduction from baseline (29). Our results demonstrated that combination therapy with ATM significantly enhanced the bactericidal activity of CZA, MEV, and IMR against all three tested strains, including *C. freundii* WYM, *C. freundii* ZGQ, and *E. coli* J53 containing pWYM_NDM_KPC (Fig. 3). Notably, ATM-based combinations achieved a ≥3 log_10_ CFU/mL reduction within 8 h, indicating rapid and potent bactericidal effects. These findings strongly suggest a synergistic interaction between ATM and the tested β-lactams/β-lactamase inhibitor combinations, resulting in significantly improved killing kinetics compared with monotherapy (Fig. 3).

## DISCUSSION

Here, we describe the genomic characteristics of two pan-drug-resistanct *C. freundii* isolates and investigated the evolution mechanism of a hybrid plasmid carrying dual carbapenem-resistance genes. Furthermore, *in vitro* experiments were conducted to explore potential therapeutic strategies against these pan-drug-resistant strains.

The co-production of multiple carbapenemases by *Enterobacteriales* poses a significant threat to public health and clinical treatment. Co-production of carbapenemases can confer resistance to new β-lactam/β-lactamase inhibitor combinations, thereby limiting the range of effective treatment options (30). The management of infections caused by multi-carbapenemase-producing *Enterobacteriales* often requires longer hospital stays, more expensive antibiotics, and additional infection control measures (31). Our study confirmed that the two isolates produced both the KPC-2 and NDM-1 carbapenemases. Notably, there are very few studies reporting the presence of fusion plasmids like pNDM_KPC; however, our findings revealed the simultaneous existence of three distinct plasmids in *C. freundii* isolates.

In a parallel study, Li et al. identified a KPC-2 and NDM-1 co-producing *C. freundii* isolate from the influx mainstream of the sewage (7). We observed the successful transfer of these plasmids to recipient cells, emphasizing not only the ease of resistance dissemination but also augmenting the potential public health threat. Similarly, Qiao et al. reported an IMP-4, NDM-1, and KPC-2 co-producing *C. freundii* from a patient with meningococcal infection (6). This repeated identification of *C. freundii* producing multiple carbapenemases in different settings underscores the need for vigilant monitoring and intervention strategies. Intriguingly, in our study, the two genes co-existed on the same plasmid in the transconjugants, thereby conferring dual resistance to the recipient cells and demonstrating the potential for future multiple-resistance scenarios in bacterial populations.

The transferability of plasmids plays a significant role in the dissemination of antibiotic resistance. Literature reports indicate that the transfer efficiency of carbapenem-resistant plasmids varies widely, with some studies noting efficiencies ranging from 10^−7^ to 10^−2^ depending on the specific plasmid and environmental conditions (32, 33). Moreover, our analysis demonstrated significant differences in conjugation efficiency among the plasmids. The pNDM_KPC plasmid exhibited a conjugation efficiency that was second only to pKPC and significantly higher than that of pNDM. Previous studies have indicated that NDM plasmids typically exhibit higher conjugation efficiency than KPC plasmids (4); however, in our study, this enhanced transferability may be attributed to the complete transfer elements present on the pKPC plasmid, facilitating the rapid dissemination of resistance traits. The absence of oriT on the NDM plasmid likely contributes to its reduced transfer capability. The implications of these findings are concerning, as they predict a heightened risk for the spread of multidrug-resistant organisms in healthcare settings, necessitating increased vigilance and effective intervention strategies.

The analysis of whole-genome sequencing has provided invaluable insights into the evolution of carbapenem-resistant hybrid plasmids. Concurrently, mobile genetic elements (MGEs), comprising insertion sequences, transposons, and gene integrons, are frequently found in multiple copies scattered across plasmids, playing a pivotal role in the transfer of resistance genes and the evolution of these plasmids (9, 34). IS*26*, a type of insertion sequence, is particularly significant in promoting the spread of resistance in Gram-negative bacteria (35). Our study found that the formation of the hybrid plasmid was facilitated by recombination mediated by IS*26* with the assistance of integron *IntI* around *bla*_KPC-2_. This IS*26*-mediated plasmid recombination is a well-reported phenomenon, especially in CRKP (35). The emerging role of IS*26* in the plasmid evolution of *C. freundii* has been noted recently (36). Reports cite instances of plasmid fusion in various ST107 *C. freundii* strains from two patients, brought about by Tn*5403*-based insertions and deletions driven by IS*26* (37). Despite the frequent involvement of IS*26* in plasmid integration and recombination events, direct IS*26*-mediated recombination between two different carbapenem resistance plasmids has been less commonly observed in the literature. Our results provide direct evidence of the formation of a dual carbapenemase-producing pathogen and the role of IS*26* in the formation of a hybrid plasmid, contributing positively to research in this area. Canonical target site duplications (TSDs) flanking IS*26* elements were not detected in both of our studied plasmids, suggesting that homologous recombination between adjacent IS*26* copies, rather than transposase-mediated excision, may underlie the observed genomic rearrangements. Besides IS*26*, the role of integrons in the recombination process cannot be overstated. Integrons are mobile genetic elements that play a pivotal role in the spread of antibiotic resistance (38). The ability of integrons to facilitate gene exchange between different plasmids can lead to the rapid emergence of multidrug-resistant bacteria, posing a significant challenge to clinical treatment and infection control (39).

While our study focused on validating the *in vitro* inhibitory efficacy of aztreonam (ATM)-based combination therapy against *Citrobacter freundii *co-producing KPC and NDM carbapenemases, these findings align with emerging preclinical evidence demonstrating therapeutic promise. Notably, Lu et al. recently reported in a murine infection model that ATM-ceftazidime/avibactam (CZA) combination therapy significantly improved survival outcomes (40), suggesting the promising efficacy of ATM-based combinations.

In conclusion, this study highlights the alarming presence of *C. freundii* isolates co-producing KPC and NDM carbapenemases, notably through the identification of the fusion plasmid pNDM_KPC. The observed transconjugation capabilities among the plasmids, particularly the high transfer efficiency of pKPC and pNDM_KPC, underscore the potential for the rapid dissemination of resistance genes. Furthermore, the role of IS*26* as a critical hotspot for plasmid integration and disintegration suggests a complex mechanism driving the evolution of these resistance traits. Our findings emphasize the urgent need for enhanced surveillance and infection control measures to combat the spread of *C. freundii* isolates co-producing KPC and NDM carbapenemases.

## Data Availability

The nucleotide sequences were submitted to the NCBI database with BioSample accession numbers SAMN35300165 and SAMN35301134.

## SUPPLEMENTAL MATERIAL

Fig. S1 spectrum.00905-25-s0001.tif The result of the CGI-Test *in vitro* multiplex immunoassay.

Fig. S2 spectrum.00905-25-s0002.tif Conjugation transfer capability predicted by oriTfinder2.

Fig. S3 spectrum.00905-25-s0003.tif Proposed formation process of the fusion plasmid of pNDM_KPC.

Fig. S4 spectrum.00905-25-s0004.tif Comparison of relative growth rates among isolates carrying carbapenem-resistant plasmids.

Supplemental material spectrum.00905-25-s0005.docx Supplemental figure legends.

Supplemental tables spectrum.00905-25-s0006.doc Tables S1 to S5.

ASM does not own the copyrights to Supplemental Material that may be linked to, or accessed through, an article. The authors have granted ASM a non-exclusive, world-wide license to publish the Supplemental Material files. Please contact the corresponding author directly for reuse.

## References

[1] Theuretzbacher U. 2013. Global antibacterial resistance: the never-ending story. J Glob Antimicrob Resist 1:63–69. doi:10.1016/j.jgar.2013.03.010 27873580

[2] Lee C-R, Lee JH, Park KS, Kim YB, Jeong BC, Lee SH. 2016. Global dissemination of carbapenemase-producing Klebsiella pneumoniae: epidemiology, genetic context, treatment options, and detection methods. Front Microbiol 7:895. doi:10.3389/fmicb.2016.00895 27379038 PMC4904035

[3] Shirley M. 2018. Ceftazidime-avibactam: a review in the treatment of serious gram-negative bacterial infections. Drugs 78:675–692. doi:10.1007/s40265-018-0902-x 29671219

[4] Gao H, Liu Y, Wang R, Wang Q, Jin L, Wang H. 2020. The transferability and evolution of NDM-1 and KPC-2 co-producing Klebsiella pneumoniae from clinical settings. EBioMedicine 51:102599. doi:10.1016/j.ebiom.2019.102599 31911273 PMC6948161

[5] Hu R, Li Q, Zhang F, Ding M, Liu J, Zhou Y. 2021. Characterisation of blaNDM-5 and blaKPC-2 co-occurrence in K64-ST11 carbapenem-resistant Klebsiella pneumoniae. J Glob Antimicrob Resist 27:63–66. doi:10.1016/j.jgar.2021.08.009 34482020

[6] Qiao J, Chen Y, Ge H, Xu H, Guo X, Liu R, Li C, Chen R, Gou J, Chen M, Zheng B. 2023. Coexistence of blaIMP-4, blaNDM-1 and blaOXA-1 in blaKPC-2-producing Citrobacter freundii of clinical origin in China. Front Microbiol 14:1074612. doi:10.3389/fmicb.2023.1074612 37378293 PMC10291173

[7] Li Y, Fang C, Qiu Y, Dai X, Zhang L. 2022. Genomic characterization of a carbapenem-resistant Citrobacter freundii cocarrying blaKPC-2 and blaNDM-1. J Glob Antimicrob Resist 29:289–292. doi:10.1016/j.jgar.2022.04.014 35489677

[8] Magobo RE, Ismail H, Lowe M, Strasheim W, Mogokotleng R, Perovic O, Kwenda S, Ismail A, Makua M, Bore A, Phayane R, Naidoo H, Dennis T, Ngobese M, Wijnant W, Govender NP. 2023. Outbreak of NDM-1– and OXA-181–producing Klebsiella pneumoniae bloodstream infections in a Neonatal Unit, South Africa. Emerg Infect Dis 29:1531–1539. doi:10.3201/eid2908.230484 37486166 PMC10370860

[9] Han X, Zhou J, Yu L, Shao L, Cai S, Hu H, Shi Q, Wang Z, Hua X, Jiang Y, Yu Y. 2024. Genome sequencing unveils blaKPC-2-harboring plasmids as drivers of enhanced resistance and virulence in nosocomial Klebsiella pneumoniae. mSystems 9:e00924-23. doi:10.1128/msystems.00924-23 38193706 PMC10878039

[10] Tang Y, Li G, Shen P, Zhang Y, Jiang X. 2022. Replicative transposition contributes to the evolution and dissemination of KPC-2-producing plasmid in Enterobacterales. Emerg Microbes Infect 11:113–122. doi:10.1080/22221751.2021.2013105 34846275 PMC8725868

[11] Yang X, Dong N, Chan EW-C, Zhang R, Chen S. 2021. Carbapenem resistance-encoding and virulence-encoding conjugative plasmids in Klebsiella pneumoniae. Trends Microbiol 29:65–83. doi:10.1016/j.tim.2020.04.012 32448764

[12] Ariyoshi T, Aoki K, Kubota H, Sadamasu K, Ishii Y, Tateda K. 2022. Molecular characterization of blaNDM-carrying IncX3 plasmids: blaNDM-16b likely emerged from a mutation of blaNDM-5 on IncX3 plasmid. Microbiol Spectr 10:e01449-22. doi:10.1128/spectrum.01449-22 35867355 PMC9430178

[13] Tian D, Wang B, Zhang H, Pan F, Wang C, Shi Y, Sun Y. 2020. Dissemination of the blaNDM-5 gene via IncX3-type plasmid among enterobacteriaceae in children. mSphere 5:e00699-19. doi:10.1128/mSphere.00699-19 PMC6952193 31915216

[14] Lopez-Diaz M, Ellaby N, Turton J, Woodford N, Tomas M, Ellington MJ. 2022. NDM-1 carbapenemase resistance gene vehicles emergent on distinct plasmid backbones from the IncL/M family. J Antimicrob Chemother 77:620–624. doi:10.1093/jac/dkab466 34993543

[15] Zhu W, Wang X, Qin J, Liang W, Shen Z. 2020. Dissemination and stability of the bla NDM-5-carrying IncX3-type plasmid among multiclonal Klebsiella pneumoniae isolates. mSphere 5:e00917-20. doi:10.1128/mSphere.00917-20 PMC7643832 33148824

[16] Clinical and Laboratory Standards Institute (CLSI). 2018. M07: clinical and laboratory standards institute methods for dilution antimicrobial susceptibility tests for bacteria that grow aerobically. Wayne, PA, USA Clinical and Laboratory Standards Institute (CLSI)

[17] Weinstein MP, Lewis JS. 2020. The Clinical and Laboratory Standards Institute Subcommittee on Antimicrobial Susceptibility Testing: background, organization, functions, and processes. J Clin Microbiol 58:e01864-19. doi:10.1128/JCM.01864-19 31915289 PMC7041576

[18] Chen Y, Yang R, Guo P, Liu P, Deng J, Wu Z, Wu Q, Huang J, Liao K. 2023. Dynamic evolution of ceftazidime–avibactam resistance due to interchanges between blaKPC-2 and blaKPC-145 during treatment of Klebsiella pneumoniae infection. Front Cell Infect Microbiol 13:1244511. doi:10.3389/fcimb.2023.1244511 37671146 PMC10476102

[19] Gu D, Yan Z, Cai C, Li J, Zhang Y, Wu Y, Yang J, Huang Y, Zhang R, Wu Y. 2023. Comparison of the NG-test carba 5, colloidal gold immunoassay (CGI) test, and Xpert carba-R for the rapid detection of carbapenemases in carbapenemase-producing organisms. Antibiotics (Basel) 12:300. doi:10.3390/antibiotics12020300 36830211 PMC9952068

[20] Xu Y, Song W, Huang P, Mei Y, Zhang Y, Xu T. 2022. A rapid carbapenemase genes detection method with Xpert carba-R from positive blood cultures compared with NG-test carba 5 and sequencing. Infect Drug Resist 15:7719–7725. doi:10.2147/IDR.S392035 36597457 PMC9805712

[21] Mendez-Sotelo BJ, López-Jácome LE, Colín-Castro CA, Hernández-Durán M, Martínez-Zavaleta MG, Rivera-Buendía F, Velázquez-Acosta C, Rodríguez-Zulueta AP, Morfín-Otero MDR, Franco-Cendejas R. 2023. Comparison of lateral flow immunochromatography and phenotypic assays to PCR for the detection of carbapenemase-producing gram-negative bacteria, a multicenter experience in Mexico. Antibiotics (Basel) 12:1–10. doi:10.3390/antibiotics12010096 PMC9855030 36671297

[22] Fu Y, Du X, Ji J, Chen Y, Jiang Y, Yu Y. 2012. Epidemiological characteristics and genetic structure of blaNDM-1 in non-baumannii Acinetobacter spp. in China. J Antimicrob Chemother 67:2114–2122. doi:10.1093/jac/dks192 22604448

[23] Li X, He J, Jiang Y, Peng M, Yu Y, Fu Y. 2021. Genetic characterization and passage instability of a hybrid plasmid co-harboring blaIMP-4 and blaNDM-1 reveal the contribution of insertion sequences during plasmid formation and evolution. Microbiol Spectr 9:e01577-21. doi:10.1128/Spectrum.01577-21 34908434 PMC8672901

[24] Grant JR, Enns E, Marinier E, Mandal A, Herman EK, Chen C, Graham M, Van Domselaar G, Stothard P. 2023. Proksee: in-depth characterization and visualization of bacterial genomes. Nucleic Acids Res 51:W484–W492. doi:10.1093/nar/gkad326 37140037 PMC10320063

[25] Wu W, Wang J, Zhang P, Wang N, Yuan Q, Shi W, Zhang X, Li X, Qu T. 2023. Emergence of carbapenem-resistant Enterobacter hormaechei ST93 plasmids co-harbouring blaNDM-1, blaKPC-2, and mcr-9 in bloodstream infection. J Glob Antimicrob Resist 34:67–73. doi:10.1016/j.jgar.2023.06.009 37369326

[26] Ji J, Du X, Chen Y, Fu Y, Wang H, Yu Y. 2013. In vitro activity of sulbactam in combination with imipenem, meropenem, panipenem or cefoperazone against clinical isolates of Acinetobacter baumannii. Int J Antimicrob Agents 41:400–401. doi:10.1016/j.ijantimicag.2012.12.014 23410789

[27] Barry AL, Craig WA, Nadler H, Reller LB, Sanders CC, Swenson JM. 1999. M26-A methods for determining bactericidal activity of antimicrobial agents; approved guideline. Vol. 19. Clinical and Laboratory Standards Institute

[28] Biagi M, Wu T, Lee M, Patel S, Butler D, Wenzler E. 2019. Searching for the optimal treatment for metallo- and serine-β-lactamase producing enterobacteriaceae: aztreonam in combination with ceftazidime-avibactam or meropenem-vaborbactam. Antimicrob Agents Chemother 63:e01426-19. doi:10.1128/AAC.01426-19 31570403 PMC6879249

[29] Smith JR, Barber KE, Raut A, Aboutaleb M, Sakoulas G, Rybak MJ. 2015. β-Lactam combinations with daptomycin provide synergy against vancomycin-resistant Enterococcus faecalis and Enterococcus faecium. J Antimicrob Chemother 70:1738–1743. doi:10.1093/jac/dkv007 25645208 PMC4542582

[30] van Duin D, Bonomo RA. 2016. Ceftazidime/avibactam and ceftolozane/tazobactam: second-generation β-lactam/β-lactamase inhibitor combinations. Clin Infect Dis 63:234–241. doi:10.1093/cid/ciw243 27098166 PMC4928383

[31] Nordmann P, Poirel L. 2019. Epidemiology and diagnostics of carbapenem resistance in gram-negative bacteria. Clin Infect Dis 69:S521–S528. doi:10.1093/cid/ciz824 31724045 PMC6853758

[32] Hardiman CA, Weingarten RA, Conlan S, Khil P, Dekker JP, Mathers AJ, Sheppard AE, Segre JA, Frank KM. 2016. Horizontal transfer of carbapenemase-encoding plasmids and comparison with hospital epidemiology data. Antimicrob Agents Chemother 60:4910–4919. doi:10.1128/AAC.00014-16 27270289 PMC4958172

[33] Yang JW, Nam J-H, Lee KJ, Yoo JS. 2024. Effect of temperature on carbapenemase-encoding plasmid transfer in Klebsiella pneumoniae Microorganisms 12:1–10. doi:10.3390/microorganisms12030454 PMC10972239 38543505

[34] Partridge SR, Kwong SM, Firth N, Jensen SO. 2018. Mobile genetic elements associated with antimicrobial resistance. Clin Microbiol Rev 31:e00088–17. doi:10.1128/CMR.00088-17 30068738 PMC6148190

[35] Jin L, Wang R, Gao H, Wang Q, Wang H. 2021. Identification of a novel hybrid plasmid encoding KPC-2 and virulence factors in Klebsiella pneumoniae sequence type 11. Antimicrob Agents Chemother 65:1–6. doi:10.1128/AAC.02435-20 PMC8315960 33722891

[36] Weber RE, Pietsch M, Frühauf A, Pfeifer Y, Martin M, Luft D, Gatermann S, Pfennigwerth N, Kaase M, Werner G, Fuchs S. 2019. IS26-mediated transfer of bla NDM-1 as the main route of resistance transmission during a polyclonal, multispecies outbreak in a German Hospital. Front Microbiol 10:2817. doi:10.3389/fmicb.2019.02817 31921015 PMC6929489

[37] Liu H, Tu Y, He J, Xu Q, Zhang X, Mu X, Chen M, Zhou H, Li X. 2024. Emergence and plasmid cointegration-based evolution of NDM-1-producing ST107 Citrobacter freundii high-risk resistant clone in China. Int J Antimicrob Agents 63:107069. doi:10.1016/j.ijantimicag.2023.107069 38141833

[38] Mazel D. 2006. Integrons: agents of bacterial evolution. Nat Rev Microbiol 4:608–620. doi:10.1038/nrmicro1462 16845431

[39] Partridge SR, Tsafnat G, Coiera E, Iredell JR. 2009. Gene cassettes and cassette arrays in mobile resistance integrons. FEMS Microbiol Rev 33:757–784. doi:10.1111/j.1574-6976.2009.00175.x 19416365

[40] Lu G, Tang H, Xia Z, Yang W, Xu H, Liu Z, Ni S, Wang Z, Shen J. 2022. In vitro and in vivo antimicrobial activities of ceftazidime/avibactam alone or in combination with aztreonam against carbapenem-resistant enterobacterales. Infect Drug Resist 15:7107–7116. doi:10.2147/IDR.S385240 36506837 PMC9733440

