# Supplementary figures and images for: High transconjugation efficiency of fusion plasmid pNDM_KPC in carbapenem-resistant Citrobacter freundii and its formation driven by IS26-mediated integration
Source: Microbiol Spectr. 2025 Aug 14;13(10):e00905-25. doi: 10.1128/spectrum.00905-25 (PMC12502794; doi:10.1128/spectrum.00905-25)

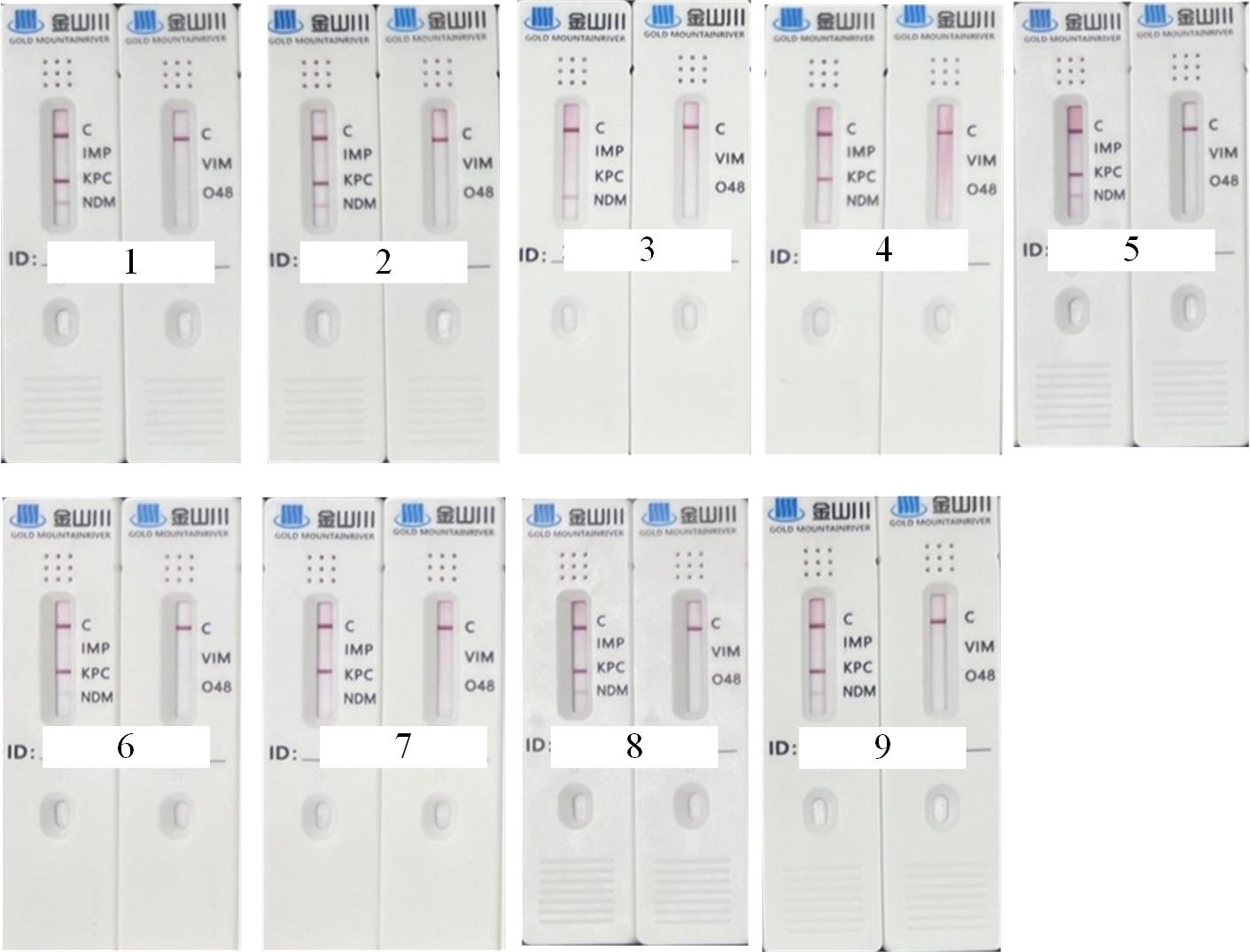

Supplement: Fig. S1 — The result of the CGI-Test in vitro multiplex immunoassay. [file spectrum.00905-25-s0001.tif]

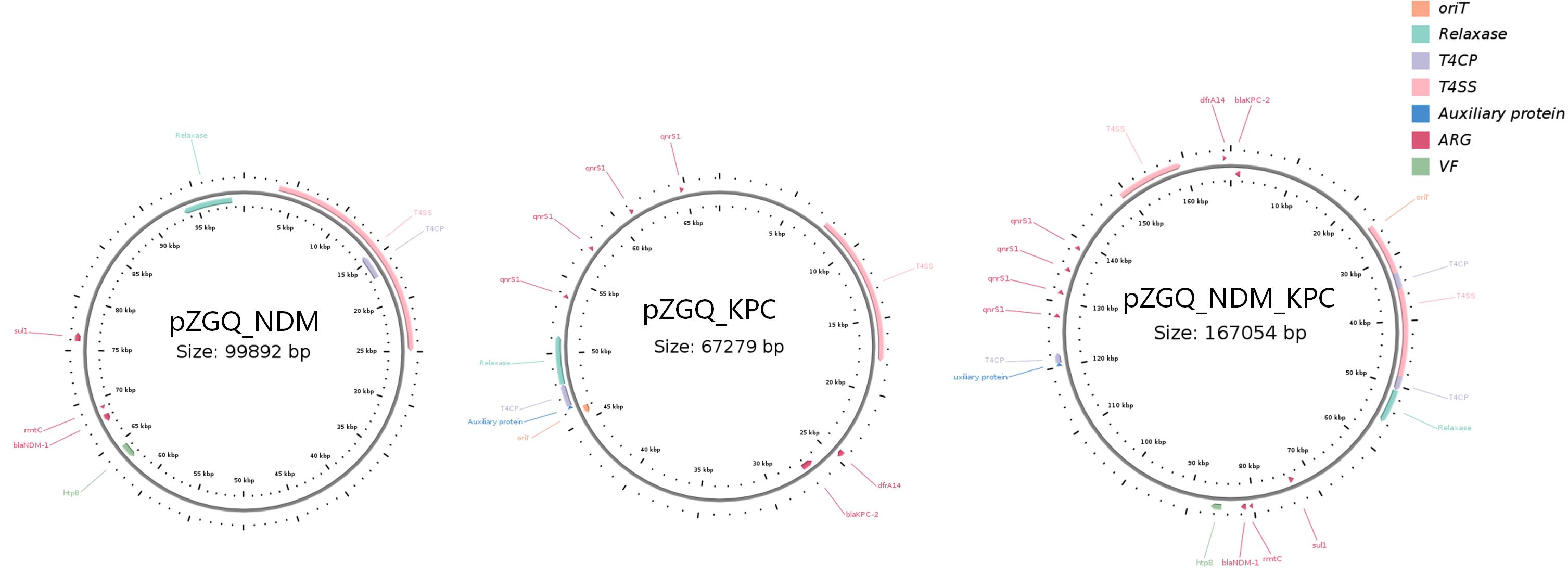

Supplement: Fig. S2 — Conjugation transfer capability predicted by oriTfinder2. [file spectrum.00905-25-s0002.tif]

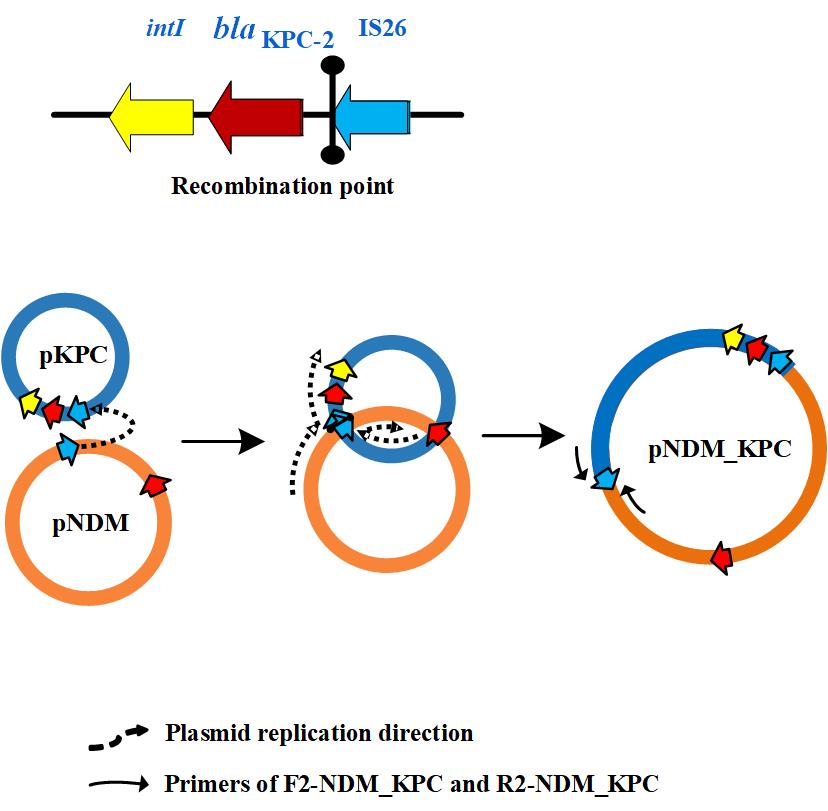

Supplement: Fig. S3 — Proposed formation process of the fusion plasmid of pNDM_KPC. [file spectrum.00905-25-s0003.tif]

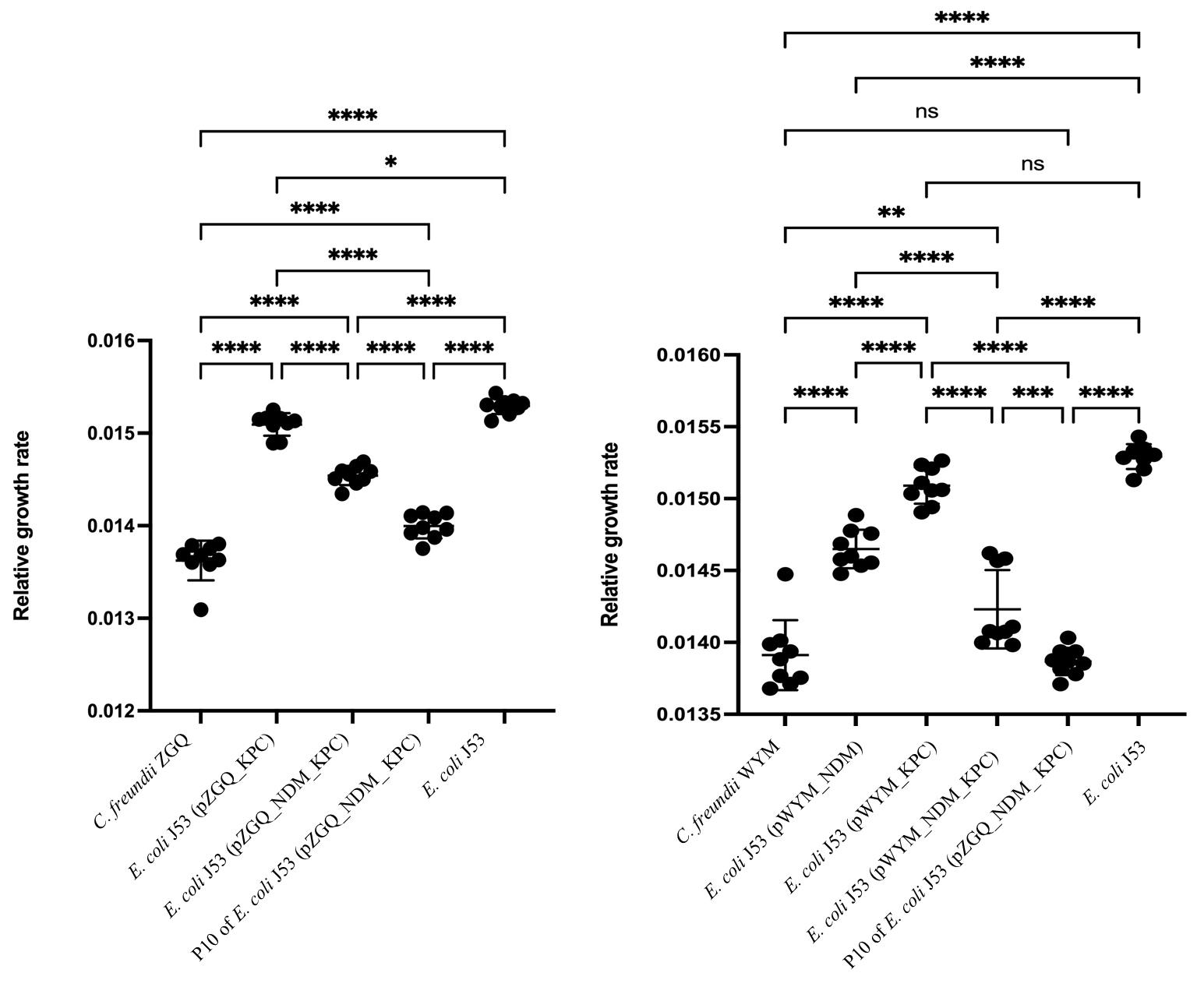

Supplement: Fig. S4 — Comparison of relative growth rates among isolates carrying carbapenem-resistant plasmids. [file spectrum.00905-25-s0004.tif]
