# Supplementary material for: High transconjugation efficiency of fusion plasmid pNDM_KPC in carbapenem-resistant Citrobacter freundii and its formation driven by IS26-mediated integration
Source: Microbiol Spectr. 2025 Aug 14;13(10):e00905-25. doi: 10.1128/spectrum.00905-25 (PMC12502794; doi:10.1128/spectrum.00905-25)
Supplement: Supplemental material — Supplemental figure legends. [file spectrum.00905-25-s0005.docx]

**Supplemental materials:**

Supplemental figure 1. The result of CGI-Test in vitro multiplex immunoassay. 1, *C. freundii* WYM; 2, *C. freundii* ZGQ; 3, *E. coli* J53 (pWYM*_*NDM); 4, *E. coli* J53 (pWYM_KPC); 5, *E. coli* J53 (pWYM_NDM_KPC); 6, *E. coli* EC600 (pWYM_NDM_KPC);7, *E. coli* J53 (pZGQ_KPC); 8, *E. coli* J53 (pZGQ_NDM_ KPC); 9, *E. coli* EC600 (pZGQ_NDM_ KPC).

Supplemental figure 2. Conjugation transfer capability predicted by oriTfinder2.

Supplemental figure 3. Proposed formation process of the fusion plasmid of pNDM_KPC. The model depicts a hypothetical mechanism for the generation of a hybrid plasmid containing both *bla*_KPC-2_ or *bla*_NDM-1_ genes, mediated by IS*26*-driven homologous recombination. The blue plasmid represents the *bla*_KPC_-carrying plasmid, while the orange plasmid represents the *bla*_NDM_ plasmid. Blue arrows indicate IS*26* elements, with yellow-filled green arrows representing intergrate sequence *IntI*. Red arrows denote the positions of the resistance genes. The formation of the hybrid plasmid is illustrated by the recombination event between the two plasmids, facilitated by the homologous IS*26* elements.

Supplemental figure 4. Comparison of relative growth rates among isolates carrying carbapenem-resistant plasmids. ns, no significant difference. *, < 0.1; **, < 0.01; ***, < 0.001; ****, < 0.0001.

**Supplemental tables:**

Supplemental table 1. Primers used for fusion plasmid of pNDM_KPC confirmation.

Supplemental table 2. Basic information of patient

Supplemental table 3. The MICs of antimicrobial susceptibility testing (AST) of donors, recipients and transconjugants (μg/mL)

Supplemental table 4. Genomics information of strains reported here.

Supplemental table 5. Strains used in stability examination of the plasmid during passage.
