# Supplementary material for: High transconjugation efficiency of fusion plasmid pNDM_KPC in carbapenem-resistant Citrobacter freundii and its formation driven by IS26-mediated integration
Source: Microbiol Spectr. 2025 Aug 14;13(10):e00905-25. doi: 10.1128/spectrum.00905-25 (PMC12502794; doi:10.1128/spectrum.00905-25)
Supplement: Supplemental tables — Tables S1 to S5. [file spectrum.00905-25-s0006.doc]

Supplemental table 1. Primers used for fusion plasmid of pNDM_KPC confirmation.

| Name | Sequence (5'->3') | Length （bp） | Target |
| --- | --- | --- | --- |
| F2-NDM_KPC | AAGCCTATTCGCTGGCTGTC | 1087 | PCR amplification |
| R2-NDM_KPC | GCCGCTACGTGAGGTCTGAT |
| Seq-up | CAACGTGAAGAAGTGGCAGA | / | Sequence primer |
| Seq-dw | CGTAAGCCGTCTTCATGGAT |

Supplemental table 2. Basic clinical information of patients.

| Patient | Specimen | Gender | Age | Ward | Diagnosis | Risk factor |
| --- | --- | --- | --- | --- | --- | --- |
| *C. freundii* WYM | Urine sample | F | 62 | Oncology | Secondary malignant breast tumors, Urine track infection | Urinary catheter |
| *C. freundii* ZGQ | Abdominal drainage | M | 50 | General Surgery | Respiratory failure, Multiple organ failure, Abdominal infection | Abdominal drainage catheter |

Supplemental table 3. The MICs of antimicrobial susceptibility testing (AST) of donors, recipients and transconjugants (μg/mL)

| Group | Strain No. | MICs (μg/mL) * | | | | | | | | | | | | | | | | |
| --- | --- | --- | --- | --- | --- | --- | --- | --- | --- | --- | --- | --- | --- | --- | --- | --- | --- | --- |
|  |  | CZO | CAZ | CTT | CRO | FEP | IMP | ERP | ATM | CIP | GEN | AMK | LEV | SXT | TOB | CZA | CO | TGC |
| Donors | *C. freundii* WYM | **>=64** | **>=64** | **>=64** | **>=64** | **>=64** | **>=16** | **>=8** | **>=64** | **>=4** | **>=16** | **>=64** | **>=8** | **>=320** | **>=16** | **>=256** | 0.5 | 1 |
|  | *C. freundii* ZGQ | **>=64** | **>=64** | **>=64** | **>=64** | **>=64** | **>=16** | **>=8** | **>=64** | **>=4** | **>=16** | **>=64** | **>=8** | **>=320** | **>=16** | **>=256** | 0.5 | 2 |
| Recipients | *E. coli* J53 | <=4 | <=1 | <=4 | <=1 | <=1 | <=1 | <=0.5 | <=1 | <=0.25 | <=1 | <=2 | <=0.25 | <=20 | <=1 | 0.5 | 0.25 | 1 |
|  | *E. coli* EC600 | <=4 | <=1 | <=4 | <=1 | <=1 | <=1 | <=0.5 | <=1 | <=0.25 | <=1 | <=2 | 0.5 | <=20 | <=1 | 0.5 | 0.5 | 0.5 |
| Transconjugants | *E. coli* J53 (pWYM_NDM) | **>=64** | **>=64** | 32 | **>=64** | 8 | **>=16** | **4** | <=1 | 1 | **>=16** | **>=64** | 1 | <=20 | **>=16** | **>=256** | 0.25 | 1 |
|  | *E. coli* J53 (pWYM_KPC) | **>=64** | **16** | <=4 | **>=64** | 2 | **>=16** | **>=8** | **>=64** | 1 | <=1 | <=2 | 1 | <=20 | <=1 | 0.5 | 0.25 | 1 |
|  | *E. coli* J53 (pWYM_ NDM_KPC) | **>=64** | **>=64** | **>=64** | **>=64** | **>=64** | **>=16** | **>=8** | **>=64** | 1 | **>=16** | **>=64** | 1 | **>=320** | >=16 | **>=256** | 0.5 | 2 |
|  | *E. coli* EC600 (pWYM_NDM_KPC) | **>=64** | **>=64** | **>=64** | **>=64** | **>=64** | **>=16** | **>=8** | **>=64** | **>=4** | **>=16** | **>=64** | **>=8** | **>=320** | >=16 | **>=256** | 0.25 | 1 |
|  | *E. coli* J53 (pZGQ_KPC) | **>=64** | **16** | <=4 | **>=64** | 2 | **>=16** | **4** | **>=64** | 1 | <=1 | <=2 | 1 | **80** | <=1 | 0.5 | 0.25 | 1 |
|  | *E. coli* J53 (pZGQ_ NDM _ KPC) | **>=64** | **>=64** | **>=64** | **>=64** | **>=64** | **>=16** | **>=8** | **>=64** | 1 | **>=16** | **>=64** | 1 | **>=320** | >=16 | **>=256** | 0.5 | 2 |
|  | *E. coli* EC600 (pZGQ_NDM_KPC) | **>=64** | **>=64** | **>=64** | **>=64** | **>=64** | **>=16** | **>=8** | **>=64** | **>=4** | **>=16** | **>=64** | **>=8** | **>=320** | >=16 | **>=256** | 0.5 | 1 |

CZO, cefazolin; CAZ, ceftazidime; CTT, cefotetan; CRO, ceftriaxone; FEP, cefepime; IMP, imipenem; ETP, ertapenem; ATM, aztreonam; CIP, ciprofloxacin; GEN, gentamicin; AMK, amikacin; LEV, levofloxacin; SXT, trimethoprim/sulfamethoxazole; TOB, tobramycin; CZA, ceftazidime/ avibactam; CO, colistin; TGC, tigecycline.

* Numbers in bold means resistance. AST was completed by using broth dilution method and the results were explained by following the breakpoint of CLSI M100 guideline.

Supplemental table 4. Genomics information of strains reported here.

| Strain No. | Location | Name | Size (bp) | ST/ plasmid typeing | GenBank accession number | Resistance genes (Copy number) | *IS*26 copies | Integrons | Transposons |
| --- | --- | --- | --- | --- | --- | --- | --- | --- | --- |
| C. freundii WYM | Chromosome | / | 5,128,115 | ST118 | CP128207 | *aac(6')-Ib-cr_1, aadA1, dfrA1, qnrS1, arr-3, bla*_CMY-48_, *catA1* | 1 | *In*2-10, *In*498 | Tn6292, Tn2012, Tn7, Tn9-like |
|  | Plasmid | pWYM_1 | 289,834 | *Inc*HI2 (smr0018), *Inc*HI2 (smr0199) | CP128208 | *terW*, *terZ* | ND | ND | *Tn*602 |
|  |  | pWYM_2 | 106,493 | ND | CP128209 | fosA3, qnrS1 (2) | 5 | ND | Tn6292 |
|  |  | pWMY_NDM | 99,892 | *Inc*FB, *Inc*FIIY | CP128210 | ble-MBL, bla_NDM-1_, rmtC, sul1 | 1 | ND | *Tn*6292, *Tn*602 |
|  |  | pWMY_KPC | 67,279 | *Inc*N | CP128211 | qnrS1 (4), dfrA14, bla_KPC-2_ | 9 | *IntI* | Tn6292 |
|  |  | pWYM_NDM_KPC | 167,054 | *Inc*FB, *Inc*FIIY, *Inc*N | / | ble-MBL, bla_NDM-1_, rmtC, sul1, qnrS1 (4), dfrA14, bla_KPC-2_ | 10 | *IntI* | Tn6292, *Tn*602 |
|  |  | pWYM_6 | 11,263 | ND | CP128213 | ND | ND | ND | ND |
|  |  | pWYM_7 | 14,755 | ND | CP128212 | ND | ND | ND | ND |
| C. freundii ZGQ | Chromosome | / | 5,128,359 | ST118 | SAMN35300165 | aadA1, aac(6')-Ib-cr, aac(6')-Ib3, dfrA1, qnrS1, arr-3, bla_CMY-48_, catA1 | 1 | *In*2-10, *In*498 | Tn6292, Tn2012, Tn7, Tn9-like |
|  | Plasmid | pZGQ_1 | 14,750 | *Inc*N (partial) | SAMN35300165 | ND | ND | ND | ND |
|  |  | pZGQ_2 | 289,796 | *Inc*HI2 (smr0018), *Inc*HI2 (smr0199) | SAMN35300165 | *terW*, *terZ* | ND | ND | *Tn*602 |
|  |  | pZGQ_NDM | 99,880 | *Inc*FB, *Inc*FIIY | SAMN35300165 | ble-MBL, bla_NDM-1_, rmtC, sul1 | 1 | ND | *Tn*6292, *Tn*602 |
|  |  | pZGQ_KPC | 67,041 | *Inc*N | SAMN35300165 | qnrS1 (4), dfrA14, bla_KPC-2_ | *9* | *In718* | Tn6292 |
|  |  | pWYM_NDM_KPC | 167,054 | *Inc*FB, *Inc*FIIY, *Inc*N | / | ble-MBL, bla_NDM-1_, rmtC, sul1, qnrS1 (4), dfrA14, bla_KPC-2_ | 10 | *IntI* | Tn6292, *Tn*602 |

ND, not detected.

Supplemental table 5. Strains used in stability examination of the plasmid during passage.

| Name | Description |
| --- | --- |
| *E. coli* J53 (pZGQ_NDM_KPC) | Tansconjugant, *E. coli* J53 containing *C. freundii* ZGQ being positive with both *bla*_KPC-2_ and *bla*_NDM-1_ on selective plates containing sodium azide and ceftazidime/avibactam. |
| *E. coli* J53 (pWYM_NDM_KPC) | Tansconjugant, *E. coli* J53 containing *C. freundii* WYM being positive with both *bla*_KPC-2_ and *bla*_NDM-1_ on selective plates containing sodium azide and ceftazidime/avibactam. |
| *E. coli* EC600 (pZGQ_NDM_KPC) | Tansconjugant, *E. coli* EC600 containing *C. freundii* ZGQ being positive with both *bla*_KPC-2_ and *bla*_NDM-1_ on selective plates containing rifampicin and ceftazidime/avibactam. |
| *E. coli* EC600 (pWYM_NDM_KPC) | Tansconjugant, *E. coli* EC600 containing *C. freundii* WYM being positive with both *bla*_KPC-2_ and *bla*_NDM-1_ on selective plates containing rifampicin and ceftazidime/avibactam. |
| *E. coli* J53 (pWYM_KPC) | Tansconjugant, *E. coli* J53 containing *C. freundii* WYM being positive with *bla*_KPC-2_ on selective plates containing sodium azide and ampicillin. |
| *E. coli* J53 (pZGQ_KPC) | Tansconjugant, *E. coli* J53 containing *C. freundii* ZGQ being positive with *bla*_KPC-2_ on selective plates containing sodium azide and ampicillin. |
| P10 of *E. coli* J53 (pZGQ_ KPC_NDM) | Single colony being positive with both *bla*_KPC-2_ gene and *bla*_NDM-1_ gene, selected from population of *E. coli* J53 (pZGQ_ NDM _ KPC) after ten days of passage in LB broth without antibiotic. |
| P10 of *E. coli* J53 (pWYM_NDM_KPC) | Single colony being positive with both *bla*_KPC-2_ gene and *bla*_NDM-1_ gene,,selected from population of *E. coli* J53 (pWYM_ NDM_KPC) after ten days of passage in LB broth without antibiotic. |
| *E. coli* J53 | Lab strain *E. coli* J53, recipient used for transconjugation experiment,sodium azide-resistant. |
| *E. coli* EC600 | Lab strain *E. coli* EC600, recipient used for transconjugation experiment, rifampicin-resistant. |
